# Identifying Student Subgroups as a Function of School Level Attributes: A Multilevel Latent Class Analysis

**DOI:** 10.3389/fpsyg.2021.624221

**Published:** 2021-02-26

**Authors:** Georgios D. Sideridis, Ioannis Tsaousis, Khaleel Al-Harbi

**Affiliations:** ^1^Boston Children’s Hospital, Harvard Medical School, Boston, MA, United States; ^2^National and Kapodistrian University of Athens, Athens, Greece; ^3^Department of Psychology, University of Crete, Rethymno, Greece; ^4^Educational Testing and Evaluation Committee, Riyadh, Saudi Arabia

**Keywords:** multilevel latent class analysis, multilevel mixture modeling, measurement invariance, cross sectional design, national data

## Abstract

The purpose of the present study was to profile high school students’ achievement as a function of their demographic characteristics, parent attributes (e.g., education), and school behaviors (e.g., number of absences). Students were nested within schools in the Saudi Arabia Kingdom. Out of a large sample of 500k, participants involved 3 random samples of 2,000 students measured during the years 2016, 2017, and 2018. Randomization was conducted at the student level to ensure that all school units will be represented and at their respective frequency. Students were nested within 50 high schools. We adopted the multilevel latent profile analysis protocol put forth by Schmiege et al. (2018) and Mäkikangas et al. (2018) that account for nested data and tested latent class structure invariance over time. Results pointed to the presence of a 4-profile solution based on BIC, the Bayes factor, and several information criteria put forth by Masyn (2013). Latent profile separation was mostly guided by parents’ education and the number of student absences (being positive and negative predictors of high achievement classes, respectively). Two models tested whether the proportions of level 1 profiles to level 2 units are variable and whether level 2 profiles vary as a function of level 1 profiles. Results pointed to the presence of significant variability due to schools.

## Introduction

Academic achievement is a very important indicator of future success in the society since academically successful individuals are more likely to have better employment opportunities, to gain higher salaries, and experience higher levels of life satisfaction and better social relationships (Rumberger and Lamb, 2003; Kuncel et al., 2004; Archambault et al., 2009; Lewis et al., 2011; Flashman, 2012; Mishook et al., 2012; Kell et al., 2013; Baroody et al., 2016; Lansford et al., 2016; Liu et al., 2020). Although there is a lack of consensus in the education literature regarding the definition of academic achievement, most scholars agree that academic achievement could be defined as the attained success in any educational act (Simpson and Weiner, 1989), or as the ability of an individual to reach a set goal through effort, skill or courage within the school context (Hornby, 2006). Over the past decades, academic achievement has been commonly assessed using school grades (i.e., GPA), standardized tests (e.g., SAT, GMAT, PISA, TIMSS, etc.), or even informal, unstandardized tests (e.g., Curriculum-based measures). Notwithstanding academic achievement’s predictive validity has been confirmed in past studies. Additionally, important correlates of academic achievement have been identified De Raad and Schouwenburg, 1996. What is less known, however, is how academic achievement and its correlates are combined to inform subgroups of students. Identification of subgroups of high school students which are associated with desirable academic outcomes may inform unique pathways to academic success. Identification of these pathways is one important endeavor but investigation of the stability of these profiles is even more important as it adds generality to the emerged subgroups. Thus, the present study targets at identifying subgroups of high school students who display adaptive school behaviors and achievement using an optimal set of school indicators proposed in the literature (see next section) and contribute information related to the consistency of these profiles over a 3-year period. Initially predictors of academic achievement are identified followed by a brief review of studies that targeted at identifying emergent student profiles. The need to identify subgroups of students in which important school and home behaviors are combined that are predictive of academic achievement can inform intervention pathways, provide educational recommendations and can lead to educational policy changes and mandates. The present study describes the methodology and means to achieve these goals.

### Predictors of Academic Achievement

Several studies have indicated that there are significant differences in academic achievement between males and females. For example, the PISA 2009 report (OECD, 2010) indicates that, on average, females perform better than males in reading comprehension, and males perform better than females in mathematics, with this pattern of results remaining unchanged in PISA 2012 and 2015 reports (OECD, 2016). Nevertheless, there is a growing body of evidence showing that females perform better than males in high school and they possess higher levels of motivation, aptitude, and self-regulation (e.g., Gibb et al., 2008; Voyer and Voyer, 2014; Fortin et al., 2015). Interestingly, the reported gender differences are not attributable to differences in cognitive ability, due to similar IQ scores across gender and socioeconomic background (Gibb et al., 2008; Matthews et al., 2009; Pirmohamed et al., 2017). Most scholars suggest that the cause for the observed difference in academic skills across gender can be attributed to future career plans. For example, Fortin et al. (2015) found that females are more successful at school because they are more interested in attending higher education.

Family structural factors, including family type (e.g., nuclear family, single-parent family, step-family), parent-child interactions, parental support, etc., are factors that play an important role in students’ academic success. Boyce-Rodgers and Rose (2001) concluded that parental monitoring and support were predictive of academic success in single, step, and nuclear type families. Veneziano (2003) suggested that parental involvement, especially father’s engagement, is a strong predictor of high school grades. Moreover, fathers’ education level, father’s expectations, and the nuclear family type were positive predictors of students’ performance in high school, especially for male students (Hines and Holcomb-McCoy, 2013).

Research has also highlighted the importance of family socioeconomic status (SES) in academic achievement. According to Conger and Donnellan (2007), SES is typically measured by composing information regarding parents’ educational level, occupational status, and income. A growing body of research suggests that a family’s ranking in the socioeconomic structure influences several aspects of a child’s development, including academic achievement. In one of the first meta-analytic attempts, White (1982) examined the relationship between SES and academic achievement, reviewing studies published before 1980 (1918–1975). He found a moderate relationship, with an average effect size of 0.34. Sirin (2005), in another meta-analytic study (examining studies from 1990 to 2000), also reported a medium-strength positive correlation, with an average effect size of 0.30 (95% CI: 0.28–0.29). Last, Liu et al. (2020), in a more recent meta-analytic study, examining samples exclusively from China (studies included, also reported a moderate relationship between SES and academic achievement (average *r* = 0.24). Several studies also showed that the relationship between SES and academic achievement in developing countries, especially in low-income countries, was weaker than that in developed countries (Heyneman and Loxley, 1983; OECD, 2016).

In an attempt to explain the underlying mechanism of how SES relates to academic achievement, Stephens et al. (2012) suggested that parents from high educational backgrounds usually create a stimulating environment at home that promotes intellectual development and boosts child’s confidence and self-esteem. Moreover, parents with a high educational background are more available and more supportive to their children, demonstrate higher educational aspirations for their children, and are more able to provide the necessary cultural and social capital that facilitates their children to succeed in school (Kim and Sherraden, 2011; Crede et al., 2015). The above explanation is also supported by the view of the sociocultural self-model, which suggests that family socioeconomic conditions affect the way children define themselves, which in turn, influence individuals’ performance not only at school but also in different aspects of social life (Stephens et al., 2012). Consequently, in the present study parental education was expected to be associated with positive academic gains.

Another finding that deserves attention is related to absenteeism and its effect on students’ academic performance. Chronic absenteeism is typically defined as missing 10% or more of school activities in a year (Balfanz and Byrnes, 2012; Gottfried, 2015). If a typical school year has 180 days, this means that students are missing at least 18 days in a year or 2 days per month. Henry (2007) identified several variables that predict student absenteeism, including the unsupervised time after school, drug use, parental education level, poor grades, and low educational aspirations.

A large body of research has documented the causal relationship between systematic absences at school (i.e., chronic absenteeism) and academic achievement. For example, Strickland (1998) found that absenteeism not only affect grades, but also performance in standardized assessments, and graduation rates. Additionally, Allesnworth et al. (2014), indicated that the absenteeism rate in middle school is a valid predictor of students who are at high risk for poor academic outcomes in high school. According to Schoeneberger (2012), over the long term, chronic absenteeism is related not only to poor academic performance but also to increased rates of high school dropout and increased likelihood of later anti-social behavior. On the other hand, found that students who attend more than 85% of the school activities are more successful in passing a standardized test in reading and mathematics than students who had lower levels of school attendance. Similarly, Aucejo and Romano (2016) found that a reduction in absences by 10 days per year leads to an increase of 5,5% in mathematics and 2.9% in reading. Interestingly, Foy (2005) indicated that absenteeism does not affect only absent students but also their peers. In a recent study, so and so reported that some level of absenteeism was present in 76.1% of the student population during the school year. Furthermore, students from the Middle Eastern region suggested that female students are usually high achievers compared to males (Hakami, 2020), and they also emit fewer absences compared to males (AlMakadma and Ramisetty-Mikler, 2015), despite contradictory findings; For example, in the Hakami (2020) study, female students emitted more absences. Consequently, it would be interesting to identify levels of absenteeism across gender as a means of informing the disparate research findings. Additionally, lack of parental guidance has been recently implicated as a cause of absences (Mahmoud, 2017). Furthermore, it would be interesting to decipher the role of absences in the presence of gender and parental education, given that the negative relationship between absenteeism and achievement has only been evident in male students (Hakami, 2020) and given the salient role of parental guidance on chronic absenteeism (Mahmoud, 2017).

Furthermore, absenteeism is considered a serious threat in the education sector, since it has direct consequences, not only to students but also to the school as an organization. For example, Harris (2014) reported that absences cost public schools $3.5 billion in state funding based on daily attendance between 2010/11 and 2012/13. For that, several programs and interventions (e.g., “Success Mentor Corps” and “WakeUp!”) have been designed to reduce chronic absenteeism. Balfanz and Byrnes (2012) found that in schools that took part in an intervention program for chronic absenteeism prevention the absenteeism rates were reduced significantly compared to schools that did not attend the program. Moreover, chronic absenteeism threatens school organization effectiveness (Lenhoff and Pogodzinski, 2018). When absenteeism rates are increased in a school, this may be an indication that certain factors within the school might cause this problem (e.g., ineffective teaching, poor school climate, loose school discipline, etc.). In that sense, absenteeism could be a potentially useful index of school performance, that could be used to reduce the phenomenon with school-based decisions.

Studies profiling students’ achievement and related behaviors have been increasing in recent years, partly due to the development of mixture modeling strategies and the ease with which these analyses are performed using contemporary software packages (e.g., SAS, STATA, Mplus, etc.). To this end, several studies attempted to profile student’s school behaviors with a large number focusing on academic motivation (e.g., Pastor et al., 2007), but also self-regulated learning (Bernacki et al., 2015), social functioning (DuPaul et al., 2018) and maladaptive school behaviors such as bullying (Shao et al., 2014; King et al., 2016). These studies usually focus on a single theoretical framework and indicators of a single latent variable from which profiles are extracted. This is in line of mixture modeling propositions in that a single latent variable can be modeled but the methodology is not limited to that; instead it can be extended to using linear combinations of predictors that span beyond a single latent variable. This is the practice followed in the present study.

## Importance of Study and Aims

The present study is important for several reasons. First, the inclusion of gender as a means of profiling students’ behaviors and achievement is extremely important as male and female students are educated in distinct educational environments. Consequently, gender is critical to our understanding of student profiles in the Saudi Arabia Kingdom and its potentially moderating role. Furthermore, the inclusion of student behaviors and their relevance to achievement such as the number of absences may provide information on both the magnitude of the relationship, the level of absenteeism as reflected in chronic absenteeism and the specific role of absences on regulating achievement across gender, as this relationship has proved to vary across gender (Hakami, 2020) suggesting potentially differential behavior between males and females when being absent from school (e.g., females may study more while at home, as absenteeism does not appear to be as harmful to them as in males). The purpose of the present study was to profile individuals’ achievement-related variables as a means of understanding the presence of subgroups in this specific population and their stability over time. Previous research employing variable-based approaches has provided unequivocal evidence regarding the predictive ability of student-level and school-level predictors of academic achievement such as the number of student absences, parental education, student gender but they fell short toward informing the presence of subpopulations. For example: “Are there subgroups of individuals in which the student-level variables are combined to make up specific profiles?,” “Are these profiles meaningful and do they agree in the pattern of the relationships with the variable based analyses?,” “What is the prevalence/existence of those subgroups in the population?,” “Are these profiles year-specific or invariant across years?,” and “How best to identify subgroups in the presence of nested structures?” These research questions are addressed in the present study using Multilevel Mixture Modeling (MMM). More specifically, a modified confirmatory latent class protocol developed by Schmiege et al. (2018) was applied to data from a national examination in Saudi Arabia to identify subgroups of students having common person-based and school-level based attributes and testing their stability over time.

## Materials and Methods

### Participants and Procedure

Participants were a random sample of students, *n* = 2000 from each of the years 2016, 2017, and 2018, which were selected using a three-step process. First, participants having full data were identified. Second, 200 schools were selected at random with sampling without replacement. At a third step, 10 students per school were selected randomly resulting in a random sample of 2,000 participants per year. The gender distribution was preserved as per the full sample (as the full cases dataset had 20:80 distributions for gender compared to 42:58 in the full sample). Thus, the total sample comprised of *n* = 6000 participants. Students came from 600 schools and the median number of students was 10. The random sample represented school affiliation equally as in the population with 82.7% of the students being educated in the public sector and 17.3% in the private sector. The mean age was 18.45 years (SD = 1.08). The students came from parents whose educational level had a mode of attending “intermediate school” with that value being in the middle of the distribution between being illiterate (0) and having received a Ph.D. degree (7).

### Measures

#### General Aptitude Test

The GAT is a national standardized assessment of general cognitive ability developed in the Arabic language for the Arabic population. The measure consists of 95 items assessing two general domains, namely verbal ability and quantitative ability. Within each domain, several subdomains are evaluated. In the verbal domain, these are a) *word meaning*, b) *sentence completion*, c) *analogy*, and, c) *reading comprehension*. For the assessment of the quantitative domain the subdomains are a) *arithmetic*, b) *analysis*, and c) *geometry*. The instrument produces both domain scores on the two major domains but also a global score using a methodology developed by Alqataee and Alharbi (2012). Items for all domains are dichotomous. Data on the focal variables under study, that is, gender, age, number of absences, parent education, and achievement scores on the GAT for Science Students were dichotomized after continuous distributions were z-score transformed. Consequently, the profiles are based on a mixture model with a categorical latent variable following the z-score dichotomization.

### Analytical Strategy: Mixture Modeling and Measurement Invariance

#### Latent Class Enumeration Process

Data were analyzed utilizing Multilevel Latent Class Mixture Modeling (MLCMM) to identify the presence of subgroups who share similar levels across a combination of predictor variables after accounting for the clustering effect (i.e., school-level variability) using a sandwich estimator which corrects standard errors for the non-independent of observations assumption. Historically speaking the models were developed for dichotomous variables (Lazarsfeld and Henry, 1968) and were later extended to include nominal and continuous indicators (Wolfe, 1970; Goodman, 1974; Moustaki, 1996). Individuals are classified into classes that best represent the observed patterns of responding in the data (Collins and Wugalter, 1992; Collins and Flaherty, 2002; Collins and Graham, 2002; Collins, 2006). The relationship of each indicator and the latent categorical variable (latent class) is characterized using a threshold parameter τ which is measured on the inverse of the logit scale with its probability of endorsability being estimated as follows:

(1) $$Probability\left(u=1|c=k\right)=\frac{1}{1+\text{exp}\,\left(\tau \right)}\;$$

Each individual is assigned a probability of membership in all classes (summed to 100%) and the model strives to classify individuals with the maximum degree of certainty on one class over the others as much as possible with the degree of that certainty being reflected in values of entropy (Cavanaugh, 1997; Nylund et al., 2007), which is a weighted average of individuals’ posterior probabilities of membership (Celeux and Soromenho, 1996). Initially, a one cluster solution is fit to the data (independence model) followed by models with two or more classes until optimum fit is obtained. In the present study, 1–6 class models were fit to the data. Model fit was judged using penalized statistics such as those provided by the AIC, and BIC related information criteria. Due to the large sample size the BIC, the consistent AIC (CAIC, Bozdogan, 1987), and the Approximate Weight of Evidence (AWE, Banfield and Raftery, 1993) criteria were indexed to aid the latent class enumeration process (Schwartz, 1978). The Bayesian Information Criterion (BIC, Schwartz, 1978; Finch and French, 2014; Yu and Park, 2014; Zhang et al., 2014) is estimated as follows:

(2) B⁢I⁢C=-2⁢L⁢L+d⁢log⁢(n)

The consistent AIC using the formula:

(3) C⁢A⁢I⁢C=-2⁢L⁢L+[d⁢log⁡(n)+1]

And the AWE:

(4) AWE=-2LL+2d[log(n)+1.5

Additional quantitative criteria were employed in the form of the approximate Bayes Factor (BF) which tests the relative fit between two models as follows (Masyn, 2013):

(5) BF=A,Bexp[SIC-ASIC]B

With SIC referring to the Schwarz Information Criterion (SIC) which is estimated as:

(6) S⁢I⁢C=-0.5×B⁢I⁢C

Based on Wasserman (Raftery, 1995) values greater than 10 points on the BF factor suggest that there is strong evidence that Model A is superior to Model B. Using the same logic, the approximate correct model probability index (cmP) compares all models with the sum value being 1, assuming one of the tested models is the true model, thus, it provides a relative comparative standard. It is estimated as follows:

(7) $$c\,m\,P_{A}=\frac{e\,x\,p\,\left(S\,I\,C_{A}-S\,I\,C_{m\,a\,x}\right)}{\sum_{j=1}^{J}e\,x\,p\,\left(S\,I\,C_{j}-S\,I\,C_{m\,a\,x}\right)}$$

With SIC max being the maximum SIC score of Model j under scrutiny. Statistical criteria favoring one model over another involved a significant reduction in the likelihood ratio test L^2^ when comparing nested models based on the difference likelihood ratio test using the Lo-Mendell-Rubin adjusted LRT and the Vuong-Lo-Mendell-Rubin LRT. Several observations are in order here. First, not all criteria will lead to the same conclusions. Thus, the enumeration process should involve a thoughtful evaluation of the information provided by the information criteria, inferential statistical criteria, model interpretation, and parsimony. Specifically, for the information criteria, Raftery (1995) pointed out that differences in nested models of less than 10 points are indicative of no differences between the models. All analyses were conducted using Mplus and using multiple random starts. Across all models, the best loglikelihood was replicated.

#### Testing Latent Class Separation

Given recommendations put forth by Finch and Bronk (2011) class separation was tested through constraining the item thresholds in one class to be equivalent to those of subsequent classes. A non-significant LRT test would signal a lack of separation and, thus, class overlap, that is, non-differentiation between the latent subgroups. A significant test, on the contrary, would indicate that subgroups are distinct concerning the latent trait under study. Additional evidence was provided using the Odds of Correct Classification (OCC) which is estimated as follows:

(8) $$O\,C\,C=\frac{\left(A\,v\,e\,P\,P/\left(1-A\,v\,e\,P\,P\right)\right)}{Est.ClassPRop/\left(1-Est.ClassProp\right)}$$

With AvePP being the average posterior probability and Est.Class Prop the estimated class proportions resulted from any related software (e.g., Mplus, Latent Gold, etc.). OCCs showing acceptable levels of between class separation have been defined as greater than 5 or 10 units (McLarnon and O’Neil, 2018).

#### Testing LCA Model Invariance

Recently, Schmiege et al. (2018) put forth a protocol on evaluating model invariance using Confirmatory Latent Class (CLC) analyses (Hoijtink, 2001; Finch and Bronk, 2011; Finch, 2015), extending the prior work of Masyn (2013) through applying some form of constraints using a single group or dual group approach. The reader is directed to these sources for detailed information. The Schmiege et al., protocol was modified along the following lines: Because the evaluation of the invariance of the thresholds was based on logits, which can reflect pretty unfair comparisons (for example the difference between a 95% probability and a 99% is more than 10 logits although their true difference is only 4% and both estimates reflect extremely high scores. Consequently, the comparison tests of constraints were modified to contrast probabilities, in addition to logits. To transform logits onto probabilities equation 1 was utilized. Then, these probabilities between two models were transformed using the arcsine transformation onto φ values as follows φ = 2^∗^arcsine (sqrt(prob. based τ)). The difference between the two φ values gives an estimate of the H-statistic, which is then evaluated for significance using a *z*-test assuming a standard normal. Further estimates involved controlling *p*-values for multiple comparisons and in estimating effect sizes as recommended by Cohen (1992).

In order to evaluate measurement invariance of the latent class solutions across two consecutive years, two models were employed (a) the fixed thresholds model, and, if that was too strict for the data, the (b) boundary constraints model. Both of these models were employed to provide information on homogeneity (boundary constraints) and separation (constrained thresholds). Models evaluated invariance using logits, probabilities, and adjustments for multiple test comparisons using the Benjamini-Hochberg multiple testing procedure and an FDR rate equal to the level of significance (i.e., 5%). The results are presented in the next section.

## Results

### Latent Class Enumeration Processes

#### Year 2016

**FIGURE 1:**
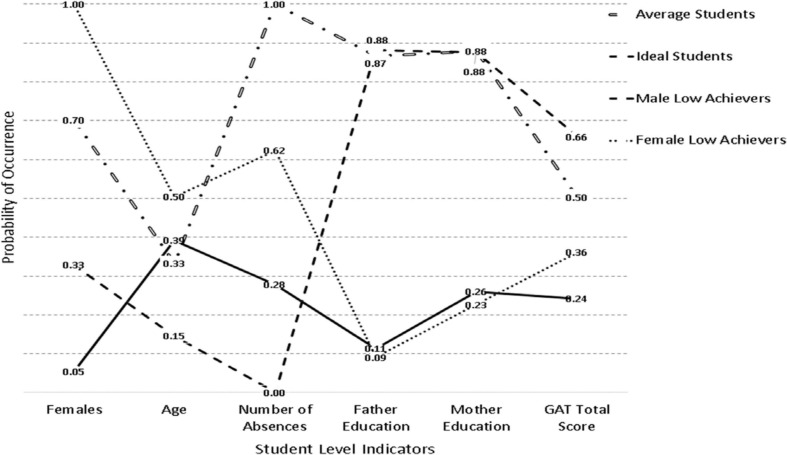
Latent class solutions in the year 2016.

Table 1 displays the findings from the class enumeration process for the year 2016. The class enumeration process is described in more detail for this year as it will represent the baseline model for which invariance would be tested across subsequent years. As shown in the table, a 4-class model seems to provide the most parsimonious fit with these data. To this conclusion corroborate the BIC and CAIC as well as the cmPK among information criteria. The AIC favored a 6-class model due to its well-knowing properties leading to favoring large models. The AWE favored a 3-class model suggesting a conservative estimate. Furthermore, both likelihood ratio tests, the VLMR and the LMR were significant when contrasting a 3-class to a 4-class model [VLMR: −2^∗^LL (7) = 70.132, *p* < 0.001; LMR: −2^∗^LL (7) = 68.838, *p* < 0.01] showing the superiority of the former but these tests were also significant in the comparison between 4 and 5 class models [VLMR: −2^∗^LL (7) = 39.341, *p* < 0.01; LMR: −2^∗^LL (7) = 38.615, *p* < 0.01] (likely showing the effects of excessive power), thus, they were not informative in the specific instance of latent class selection. Additionally, the OCCs ranged between 16 and 33 showing adequate separation in the 4-class model, with estimates in the 5-class solution being below the threshold of 10. Thus, collectively and relying on the principle of parsimony, information criteria, and ease of interpretation, a 4-class solution was the preferred choice with these data. Figure 1 displays the 4 classes, which, for ease of interpretation were termed, “Ideal Students,” “Average Students,” “Male Low Achievers,” and “Female Low Achievers.” As shown in the figure, the “ideal student” class was comprised of individuals from whom 33% were female, who were younger, emitted no absences, had very well educated parents, and achieved the highest educational outcomes based on the GAT Science test. The “Average Student” group had average achievement and their difference to the ideal student group was that they emitted many more absences, were older, and were predominantly female (70%). The “Male Low Achievers” and the “Female Low Achievers” classes were low achieving classes with students of the same age, whose parents were of very poor educational background. The only then difference between the two classes besides their gender composition was on the number of absences; the low achieving females class emitted many more absences compared to the low achieving male class. Furthermore, the classes were defined with ample participants. Specifically, the average student class had 382 participants (19.1%), the ideal class 801 participants (40.05%), the male low achievers 520 (26.0%), and the female low achievers (297 (14.85%).

**TABLE 1:** Latent class solution of student behaviors at the year 2016.

**Model**	**LL**	**npar**	**AIC**	**BIC**	**CAIC**	**AWE**	**BF (K, K+1)**	**cmP(K)**	**SIC**	**exp(SIC-max)**	**Entropy**
1-class	−7973.65	6	15959.29	15985.33	15991.33	16041.38	0.000	0.000	−7992.7	2.28E-197	-
2-class	−7609.03	13	15244.05	15300.48	15313.48	15421.90	0.000	0.000	−7650.2	1.181E-48	0.657
3-class	−7489.36	20	15018.71	15105.52	15125.52	**15292.32**	0.000	0.000	−7552.8	2.557E-06	0.800
4-class	−7454.29	27	14962.57	**15079.76**	**15106.76**	15331.95	12.632	**0.927**	−7539.9	**1**	0.803
5-class	−7434.63	34	14937.26	15084.84	15118.84	15402.41	10211.37	0.073	−7542.4	0.079161	0.763
6-class	−7421.67	41	**14925.34**	15103.30	15144.30	15486.25	0.000	0.000	−7551.6	7.752E-06	0.735

*LL = loglikelihood; npar = number of free parameters in the model; AIC = Akaike information criterion; BIC = Bayesian information criterion; CAIC = Bozdogan’s consistent AIC; AWE = Approximate weight of evidence criterion (Masyn, 2013); BF = Bayes factor; cmP(k) = correct model probability; SIC = Schwartz information criterion. Entropy estimates are presented to realize latent class separation; this information did not contribute to the class enumeration process. Bolded estimates show preference by a criterion for a given class solution.*

#### Year 2017

**FIGURE 2:**
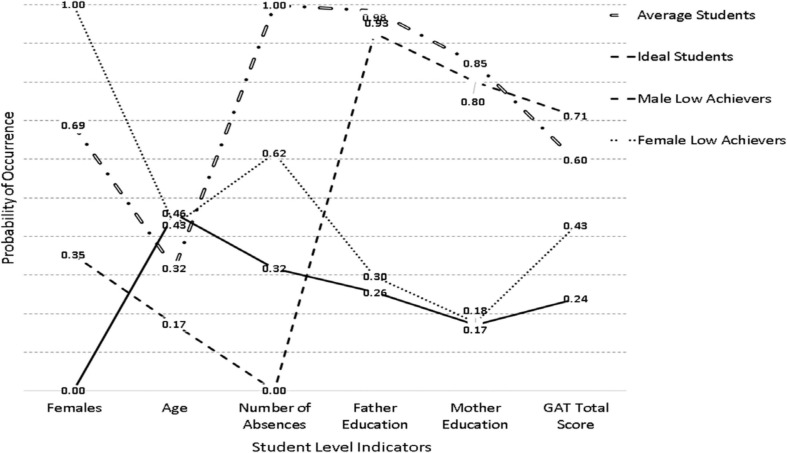
Latent class solutions in the year 2017.

Table 2 displays the respective findings from the class enumeration process for the year 2017. The purpose of this analysis was to profile the groups in the year 2017 in the absence of information in the year 2016. Subsequently, the goal was, if latent classes were of the same number, to test for the invariance of the solutions across years. As shown in Table 2, the BIC, CAIC, and cmPK favored a 4-class solution as well as the VLMR and LMR tests which favored the 4-class solution over the 3-class solution [VLMR: −2^∗^LL(7) = 85.319, *p* < 0.001; LMR: −2^∗^LL (7) = 83.745, *p* < 0.01] but failed to find differences between the 4-class and 5-class solutions [VLMR: −2^∗^LL (7) = 32.005, *p* = 0.78; LMR: −2^∗^LL ^2^(7) = 31415, *p* = 0.78]. As with year 2016, OCCs pointing to adequate separation ranged between 16 and 43. Based on those inferential tests, and for reasons of parsimony, the 4-class solution was selected. Furthermore, the AIC and AWE chose 6 and 3 classes, respectively. The 4-class model is shown in Figure 2 and poses a high resemblance to the respective subgroups emerged in the year 2016 with some notable differences. For example, the female low achieving class was of higher ability approaching the middle of the distribution compared to the respective estimates in the year 2016. Furthermore, the average student group was of higher achievement as well (from 0.50 to 0.60). Last, the classes were defined with ample participants. Specifically, the average student class had 292 participants (14.6%), the ideal class 678 participants (33.9%), the male low achievers 610 (30.5%), and the female low achievers 420 (21.0%).

**TABLE 2:** Latent class solution of student behaviors at the year 2017.

**Model**	**LL**	**npar**	**AIC**	**BIC**	**CAIC**	**AWE**	**BF (K, K+1)**	**cmP(K)**	**SIC**	**exp(SIC-max)**	**Entropy**
1-class	−8068.22	6	16148.43	16174.47	16180.47	16230.51	0.000	0.000	−8087.2	2.48E-177	-
2-class	−7718.45	13	15462.90	15519.32	15532.32	15640.75	0.000	0.000	−7759.7	4.549E-35	0.622
3-class	−7637.63	20	15315.27	15402.08	15422.08	**15588.88**	0.000	0.000	−7701	1.313E-09	0.757
4-class	−7594.99	27	15243.98	**15361.17**	**15388.17**	15613.36	489.438	**0.998**	−7680.6	**1**	0.812
5-class	−7578.99	34	15225.99	15373.56	15407.56	15691.13	1091711.890	0.002	−7686.8	0.0020432	0.763
6-class	−7570.71	41	**15223.41**	15401.37	15442.37	15784.32	0.000	0.000	−7700.7	1.872E-09	0.682

*LL = loglikelihood; npar = number of free parameters in the model; AIC = Akaike information criterion; BIC = Bayesian information criterion; CAIC = Bozdogan’s consistent AIC; AWE = Approximate weight of evidence criterion (Masyn, 2013); BF = Bayes factor; cmP(k) = correct model probability; SIC = Schwartz information criterion. Entropy estimates are presented to realize latent class separation; this information did not contribute to the class enumeration process. Bolded estimates show preference by a criterion for a given class solution.*

#### Year 2018

**FIGURE 3:**
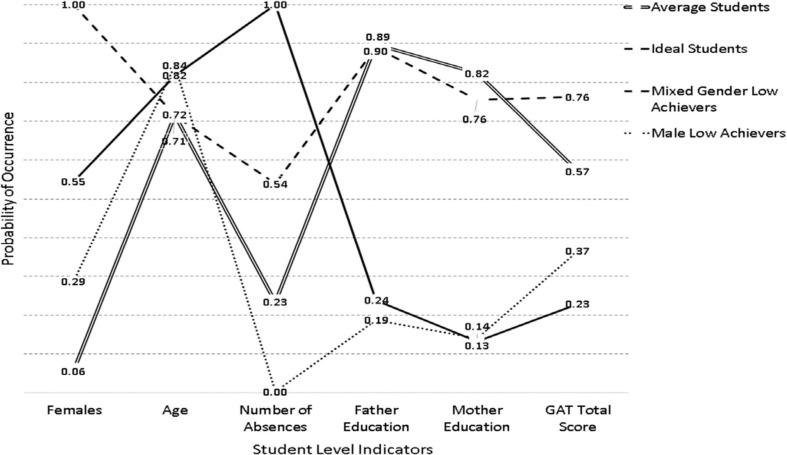
Latent class solutions in the year 2018.

The respective findings from the analysis of subgroups for the year 2018 are shown in Figure 3 and Table 3. As shown in the Table, a 4-class solution was favored by the BIC and CAIC as well as the cmPK with the AIC and AWE favoring a 6-class and a 3-class solution, respectively. The inferential tests showed that the 4-class solution was favored in comparison to the 3-class solution using both the VLMR and the LMR tests [VLMR: −2^∗^LL(7) = 64.891, *p* < 0.001; LMR: −2^∗^LL (7) = 63.694, *p* < 0.001]; however, the difference using these criteria when contrasting 4 vs. 5 classes resulted in mixed results [VLMR: −2^∗^LL(7) = 27.712, *p* = 0.04; LMR: −2^∗^LL (7) = 27.201, *p* = 0.05], with the VLMR suggesting a 5-class solution and the LMR a 4-class solution. In both 4 and 5-class solutions OCCs showed proper separation. Consequently, for reasons of parsimony, a 4-class solution was adopted here as well. Despite some resemblance, there were salient differences between these classes and the respective classes observed in the years 2016 and 2017. More specifically, these participants were older compared to previous years. Given, the negative role of age in achievement, that finding may have affected the formation of student subgroups. Other differences involved the previously termed “female low achieving group” which was now termed mixed because there was an approximately equal distribution between males and females (55% males, 45% females). Another difference was that this mixed low achieving group had students who had many more absences compared to the previously described female group whose levels of absences were much lower (0.62 in the year 2016 compared to 1.0 in the year 2018). Last, the classes were defined with ample participants. Specifically, the average achieving class had 616 participants (30.8%), the ideal class 473 participants (23.65%), the male low achievers 524 (26.20%), and the “mixed low achievers” 387 (19.35%).

**TABLE 3:** Latent class solution of student behaviors at the year 2018.

**Model**	**LL**	**npar**	**AIC**	**BIC**	**CAIC**	**AWE**	**BF (K, K+1)**	**cmP(K)**	**SIC**	**exp(SIC-max)**	**Entropy**
1-class	−7907.24	6	15826.47	15852.51	15858.51	15908.55	0.000	0.000	−7926.3	2.43E-150	-
2-class	−7598.58	13	15223.15	15279.58	15292.58	15401.00	0.000	0.000	−7639.8	6.257E-26	0.611
3-class	−7528.60	20	15097.19	15184.00	15204.00	**15370.80**	0.000	0.000	−7592	3.562E-05	0.761
4-class	−7496.16	27	15046.32	**15163.51**	**15190.51**	15415.70	4098.013	**1.000**	−7581.8	**1**	0.760
5-class	−7482.29	34	15032.58	15180.15	15214.15	15497.72	67255.73	0.000	−7590.1	0.000244	0.668
6-class	−7471.21	41	15024.43	15202.38	15243.38	15585.34	0.000	0.000	−7601.2	3.628E-09	0.657

*LL = loglikelihood; npar = number of free parameters in the model; AIC = Akaike information criterion; BIC = Bayesian information criterion; CAIC = Bozdogan’s consistent AIC; AWE = Approximate weight of evidence criterion (Masyn, 2013); BF = Bayes factor; cmP(k) = correct model probability; SIC = Schwartz information criterion. Entropy estimates are presented to realize latent class separation; this information did not contribute to the class enumeration process. Bolded estimates show preference by a criterion for a given class solution.*

### Latent Class Solution Invariance Across Years

Several models were implemented to evaluate the invariance of the latent class solutions over time using one-sample and dual sample approaches. The one-sample approach entailed imposing the same estimates of 1 year to that of another year. A significant misfit would indicate a lack of invariance and the opposite would support a conclusion of invariant measurement. These tests were also supplemented with the dual sample approach in which multigroup latent class models were fit to the data with the imposition of equality constraints across thresholds of adjacent periods.

**FIGURE 4:**
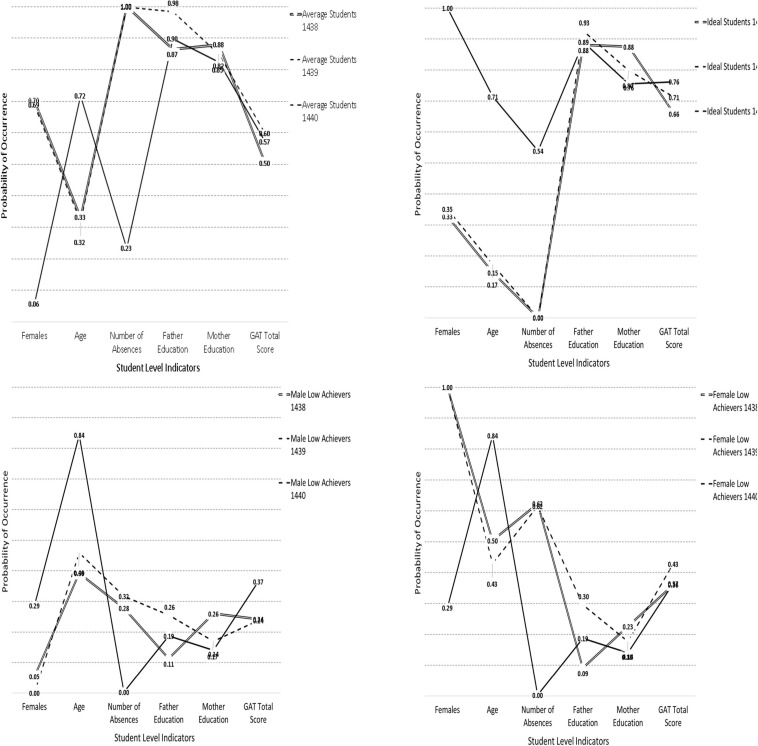
Visual analysis of latent class stability over the years for each one of the 4 latent classes.

Using the one sample approach, estimates in logits during the year 2017 were fixed to be equivalent to those of the year 2016. Likelihood ratio difference tests addressed the null hypothesis that the profiles observed in the year 2016 were invariant during the year 2017. Results indicated significant differences between profiles using the difference LRT test [Chi-square(24) = 303.944, *p* < 0.001]. When testing differences between profiles in the years 2017 and 2018, again, significant differences were observed [Chi-square(24) = 1581.873, *p* < 0.001]. Last, when comparing the years 2016 and 2018, non-invariance in the subgroup profiles was again observed [Chi-square(24) = 2033.865, *p* < 0.001]. Thus, statistically speaking, the subgroup formation varied significantly across years using independent samples. Figure 4 displays the profiles within each year to allow for a visual inspection of the differences across them over time. As shown in the figure, the solutions in the years 2016 and 2017 pose great resemblance, but the solution during the year 2018, is quite variable compared to the earlier years.

When looking at the profile of average students (Figure 4, upper left panel), it is apparent that there was substantial overlap between the years 2016 and 2017 with few discrepancies in the parent education variables and achievement. This class, however, was not observed or replicated in the year 2018 as the newly emerged class was not comprised of females and younger individuals nor was similar in the number of absences. By visual analysis, this is the biggest discrepancy in that the profile of average students was not observed in the year 2018.

When looking at the profiles of “ideal students” again, the subgroups were very similar in the years 2016 and 2017 but became discrepant during 2018. Specifically, the ideal student group during the first 2 years was comprised of mostly males whereas in the year 2018 was a predominantly female group. Furthermore, there were salient differences in the number of absences across classes with the female successful class of 2018 having high achievement despite emitting a modest amount of absences (a fact that was observed in the moderated regression analyses using the variable based approach).

The third class, the male low achieving class (see Figure 4, lower left panel), was similar across years with the again year 2018 being markedly different. What differentiated the subgroups in the year 2018 was that this group was much older, and it contained some degree of females as well. Achievement levels were also somewhat higher for that group, likely because of emitting zero absences.

Last, the “female low achieving” class was again not replicated in the year 2018 as that low achievement subgroup contained mostly males, which is why it was termed the “mixed” group in the 2018 LCA analysis. Again, the profiles for years 2016 and 2017 were very similar, despite being statistically speaking different, a fact that could be attributed to excessive power levels (e.g., the multigroup analyses were run with 6,000 participants). In an attempt to validate the findings from the inferential statistics, using a series of constrained models. The most relaxing test of invariance involved the “boundary constraints” approach. Based on Schmiege et al. (2018), this approach entails posing low bound and upper bound constraints on the values of the baseline year when logits were significantly different from zero. The actual values of the constrained estimates were the values at the 95% Confidence Intervals of the baseline model. A well-fitted model would suggest that the low bound constraints were proper, leading to a tentative conclusion of class homogeneity across years. The results from the unconstrained, fixed thresholds and boundary constraints analyses are shown in Table 4. As shown in the table, significant differences were evident when utilizing the fixed thresholds model. So, the exact fit and equivalence of the latent class solutions cannot be inferred from these data. When looking at the boundary constraints, and by use of the information criteria, models again appear to be dissimilar over time. So, the overall conclusion of a lack of invariance should be drawn when evaluating these models. In order to identify the magnitude of non-invariance, several analyses were employed. Those involved difference tests in logits, probabilities, and relevant effect size criteria. These results are shown in Tables 5–7.

**TABLE 4:** Measurement invariance of latent class solutions across years.

**Model**	**LL**	**npar**	**LR chi-sq**	**Difference Test**	**AIC**	**BIC**	**SABIC**
**Contrasting Years 2016 vs. 2017**							
Unconstrained 2017	−7594.974	27	64.403*	-	15243.947	15395.172	15309.391
Fixed Thresholds	−7750.220	3	339.437*	*p* < .001	15506.441	15523.244	15513.713
Boundary Constraints	−7602.095	24	78.634*	*p* < .001	15252.190	15386.612	15310.363
Contrasting Years 2017 vs. 2018							
Unconstrained 2018	−7496.161	27	65.503*	-	15046.321	15197.546	15111.765
Fixed Thresholds	−8350.679	3	1578.341*	*p* < .001	16707.359	16724.162	16714.630
Boundary Constraints	−7867.721	23	736.548*	p < .001	15781.442	15910.263	15837.191
**Contrasting Years 2016 vs. 2018**							
Unconstrained 2018	−7496.161	27	65.503*	-	15046.321	15197.546	15111.765
Fixed Thresholds	−8594.842	3	2006.486*	*p* < .001	17195.683	17212.486	17202.955
Boundary Constraints	−7837.721	23	536.548*	*p* < .001	15681.442	15810.263	15737.191

*LL = loglikelihood; npar = number of free parameters in the model; LRchi-sq is the likelihood ratio Chi-square test (not the Pearson one). AIC = Akaike information criterion; BIC = Bayesian information criterion; SABIC = Sample-adjusted BIC. The difference test refers to Chi-square difference tests in the presence of nested models.*

**TABLE 5:** Differences between thresholds across waves (2016 vs. 2017) using logit and probability difference tests.

**Threshold τ**	**Logit Diff**	***p*-value Logit**	**Logit Diff B-H**	**prob-Diff**	***p*-value prob.**	***p*-Diff B-H**	**H**	**H E.S. Cohen**	**H *Z*-test**	**H *Z*-test B-H**
**T1**	0.095	0.513	n.s.	−0.021	0.513	n.s.	−0.045	Small	−0.6540	n.s.
**T2**	0.267	0.132	n.s.	−0.035	0.137	n.s.	−0.097	Small	−1.4990	n.s.
**T3**	10.689	0.001*	n.s.	−0.013	0.731	n.s.	−0.230	Small	−0.6820	n.s.
**T4**	0.140	0.701	n.s.	−0.012	0.698	n.s.	−0.041	Small	−0.3860	n.s.
**T5**	−0.754	0.001*	n.s.	0.101	0.001*	n.s.	0.275	Small	3.3630	**Sig.**
**T6**	0.167	0.192	n.s.	−0.036	0.189	n.s.	−0.077	Small	−1.3100	n.s.
**T7**	−6.735	0.000*	**Sig.**	0.305	0.000*	**Sig.**	1.126	Large	10.3340	**Sig.**
**T8**	−0.718	0.002	n.s.	0.171	0.001*	n.s.	0.351	Small	3.1370	**Sig.**
**T9**	14.467	0.000*	**Sig.**	−0.370	0.000*	**Sig.**	−1.306	Large	−8.8420	**Sig.**
**T10**	5.551	0.001*	n.s.	−0.851	0.000*	**Sig.**	−2.097	Large	−7.1130	**Sig.**
**T11**	2.636	0.000*	**Sig.**	−0.569	0.000*	**Sig.**	−1.222	Large	−5.1420	**Sig.**
**T12**	0.852	0.001*	n.s.	−0.210	0.001*	n.s.	−0.423	Small	−3.3330	**Sig.**
**T13**	14.153	0.000*	**Sig.**	−0.300	0.000*	**Sig.**	−1.158	Large	−11.7950	**Sig.**
**T14**	0.466	0.019	n.s.	−0.109	0.018	n.s.	−0.225	Small	−2.3540	n.s.
**T15**	−4.854	0.127	n.s.	0.375	0.000*	**Sig.**	1.190	Large	5.1520	**Sig.**
**T16**	−3.146	0.000*	**Sig.**	0.620	0.000*	**Sig.**	1.383	Large	5.6100	**Sig.**
**T17**	−3.832	0.000*	**Sig.**	0.737	0.000*	**Sig.**	1.667	Large	9.9590	**Sig.**
**T18**	−0.333	0.061	n.s.	0.083	0.060	n.s.	0.166	Small	1.8790	n.s.
**T19**	−12.086	0.000*	**Sig.**	0.051	0.491	n.s.	0.457	Small	1.3490	n.s.
**T20**	0.331	0.032	n.s.	−0.081	0.031	n.s.	−0.164	Small	−7.1130	**Sig.**
**T21**	0.217	0.189	n.s.	−0.045	0.187	n.s.	−0.099	Small	−1.3170	n.s.
**T22**	0.711	0.019	n.s.	−0.106	0.011	n.s.	−0.274	Small	−2.4750	n.s.
**T23**	−0.738	0.003	n.s.	−0.720	0.000*	**Sig.**	−2.025	Large	−11.7950	**Sig.**
**T24**	−0.128	0.408	n.s.	−0.181	0.000*	**Sig.**	−0.385	Small	−2.3540	n.s.

*For the H *z*-test, the critical value of the z-statistic at a = 0.001 is 3.0902. Diff = Difference estimate. B-H = Benjamini-Hochberg correction. H = Statistic expressing the difference in two proportions using arcsine transformation. E.S. = Effect Size. Bold text indicates significant effects.*

**TABLE 6:** Differences between thresholds across waves (2017 vs. 2018) using logit and probability difference tests.

**Threshold τ**	**Logit Diff**	***p*-value Logit**	**Logit Diff B-H**	**prob-Diff**	***p*-value prob.**	**p-Diff B-H**	**H**	**H E.S. Cohen**	**H *Z*-test**	**H *Z*-test B-H**
**T1**	−1.245	0.008	n.s.	0.216	0.000*	**Sig.**	0.516	Medium	−2.652	n.s.
**T2**	2.470	0.000*	**Sig.**	−0.539	0.000*	**Sig.**	−1.151	Large	14.449	**Sig.**
**T3**	13.820	0.000*	**Sig.**	−0.235	0.000*	**Sig.**	−1.011	Large	13.687	**Sig.**
**T4**	−0.249	0.596	n.s.	0.020	0.597	n.s.	0.070	Small	−0.530	n.s.
**T5**	0.206	0.452	n.s.	−0.031	0.441	n.s.	−0.080	Small	0.753	n.s.
**T6**	−0.586	0.000*	**Sig.**	0.134	0.000*	**Sig.**	0.280	Small	−3.920	**Sig.**
**T7**	0.000	1.000	n.s.	0.000	1.000	n.s.	0.000	Small	0.000	n.s.
**T8**	1.220	0.000*	**Sig.**	−0.289	0.000*	**Sig.**	−0.593	Medium	6.475	**Sig.**
**T9**	−0.111	0.673	n.s.	0.027	0.674	n.s.	0.054	Small	−0.422	n.s.
**T10**	2.988	0.000*	**Sig.**	−0.602	0.000*	**Sig.**	−1.331	Large	7.044	**Sig.**
**T11**	2.672	0.000*	**Sig.**	−0.578	0.000*	**Sig.**	−1.240	Large	7.837	**Sig.**
**T12**	1.639	0.000*	**Sig.**	−0.367	0.000*	**Sig.**	−0.774	Medium	6.561	**Sig.**
**T13**	−0.453	0.115	n.s.	0.105	0.119	n.s.	0.218	Small	−1.577	n.s.
**T14**	2.196	0.000*	**Sig.**	−0.489	0.000*	**Sig.**	−1.034	Large	8.723	**Sig.**
**T15**	−3.429	0.887	n.s.	0.001	0.364	n.s.	0.061	Small	−0.142	n.s.
**T16**	−4.825	0.006	**Sig.**	0.727	0.000*	**Sig.**	1.787	Large	−2.762	n.s.
**T17**	−3.495	0.000*	**Sig.**	0.702	0.000*	**Sig.**	1.558	Large	−6.188	**Sig.**
**T18**	−1.679	0.000*	**Sig.**	0.379	0.000*	**Sig.**	0.797	Medium	−6.633	**Sig.**
**T19**	6.761	0.000*	**Sig.**	−0.287	0.000*	**Sig.**	−1.088	Large	13.087	**Sig.**
**T20**	1.846	0.000*	**Sig.**	−0.383	0.000*	**Sig.**	−0.838	Large	10.297	**Sig.**
**T21**	−1.578	0.048	n.s.	0.229	0.001	n.s.	0.595	Medium	−1.974	n.s.
**T22**	−0.328	0.258	n.s.	0.056	0.240	n.s.	0.136	Small	−1.132	n.s.
**T23**	−0.143	0.618	n.s.	−0.417	0.000*	n.s.	−0.898	Large	−0.499	n.s.
**T24**	0.615	0.000*	**Sig.**	−0.573	0.000*	**Sig.**	−1.223	Large	3.751	**Sig.**

*For the H *z*-test, the critical value of the z-statistic at a = 0.001 is 3.0902. Diff = Difference estimate. B-H. = Benjamini-Hochberg correction. H = Statistic expressing the difference in two proportions using arcsine transformation. E.S. = Effect Size. Bold text indicates significant effects.*

**TABLE 7:** Differences between thresholds across waves (2016 vs. 2018) using logit and probability difference tests.

**Threshold T**	**Logit Diff**	***p*-value Logit**	**Logit Diff B-H**	**prob-Diff**	***p*-value prob.**	***p*-Diff B-H**	**H**	**H E.S. Cohen**	**H *Z*-test**	**H *Z*-test B-H**
**T1**	14.153	0.000*	**Sig.**	−0.300	0.000*	**Sig.**	−1.158	Large	−12.509	**Sig.**
**T2**	1.656	0.000*	**Sig.**	−0.391	0.000*	**Sig.**	−0.804	Large	−8.550	**Sig.**
**T3**	−14.600	0.000*	**Sig.**	0.401	0.000*	**Sig.**	1.371	Large	13.513	**Sig.**
**T4**	−0.083	0.890	n.s.	0.009	0.889	n.s.	0.027	Small	0.139	n.s.
**T5**	−1.086	0.006	n.s.	0.157	0.002	n.s.	0.411	Small	2.974	n.s.
**T6**	1.303	0.000*	**Sig.**	−0.285	0.000*	**Sig.**	−0.608	Medium	−5.591	**Sig.**
**T7**	−5.025	0.455	n.s.	0.428	0.000*	**Sig.**	1.296	Large	2.732	n.s.
**T8**	1.534	0.000*	**Sig.**	−0.327	0.000*	**Sig.**	−0.707	Medium	−7.117	**Sig.**
**T9**	14.598	0.000*	**Sig.**	−0.401	0.000*	**Sig.**	−1.370	Large	−17.105	**Sig.**
**T10**	0.885	0.088	n.s.	−0.120	0.050	n.s.	−0.325	Small	−1.860	n.s.
**T11**	−0.871	0.022	n.s.	0.127	0.033	n.s.	0.332	Small	2.256	n.s.
**T12**	−0.772	0.003	n.s.	0.155	0.003	n.s.	0.345	Small	2.981	n.s.
**T13**	−0.975	0.007	n.s.	0.173	0.001	n.s.	0.409	Small	2.999	n.s.
**T14**	2.715	0.000*	**Sig.**	−0.573	0.000*	**Sig.**	−1.242	Large	−20.442	**Sig.**
**T15**	13.819	0.000*	**Sig.**	−0.235	0.000*	**Sig.**	−1.011	Large	−17.544	**Sig.**
**T16**	−0.080	0.816	n.s.	0.008	0.817	n.s.	0.025	Small	0.232	n.s.
**T17**	−0.603	0.021	n.s.	0.079	0.029	n.s.	0.218	Small	2.260	n.s.
**T18**	−0.396	0.004	n.s.	0.093	0.004	n.s.	0.192	Small	2.870	n.s.
**T19**	4.150	0.000*	**Sig.**	−0.282	0.000*	**Sig.**	−0.974	Large	−9.417	**Sig.**
**T20**	2.178	0.000*	**Sig.**	−0.460	0.000*	**Sig.**	−0.997	Large	−1.860	n.s.
**T21**	−1.916	0.121	n.s.	0.224	0.001	n.s.	0.643	Medium	2.285	n.s.
**T22**	0.298	0.448	n.s.	−0.036	0.463	n.s.	−0.104	Small	−0.748	n.s.
**T23**	−0.975	0.003	n.s.	0.117	0.027	n.s.	0.287	Small	2.999	n.s.
**T24**	0.529	0.001	n.s.	−0.470	0.000*	**Sig.**	−0.979	Large	−20.442	**Sig.**

*For the H *z*-test, the critical value of the z-statistic at a = 0.001 is 3.0902. Diff = Difference estimate. B-H = Benjamini-Hochberg correction. H = Statistic expressing the difference in two proportions using arcsine transformation. E.S. = Effect Size. Bold text indicates significant effects.*

### Tests for Invariance of Single Parameters: Contrasting Thresholds Using Tests of Significance and Effect Size Indicators

These tests were conducted to understand more in-depth the magnitude of non-invariance. In particular, we were interested in the difference in conclusions drawn when utilizing logits versus probabilities. The idea is simple as a difference in several logits may reflect a very small change in the probability of endorsing a specified behavior. Thus, the tests of probability may be more accurate in understanding non-invariance.

Table 5 displays the findings from contrasting the years 2016 and 2017. As shown in the table the number of significant tests using the logit was 9/24 (37.5%) and that number went down when applying an FDR correction using an alpha level of 0.001. Specifically, the number of significant tests was 7/24, that is, 29.2%. Using the probability difference tests, there were 13 significant tests, thus, non-invariance was at 54.2%, and using the *z*-test from the H-statistic (arcsine transformation) similar findings for non-invariance emerged (i.e., 12/24, 50%). When utilizing effect size conventions of the H-statistic, however, which reflects differences in proportions, there were 9 large effects (37.5%), and 15 small effects (62.5%). Thus, a general conclusion from contrasting years 2016 and 2017 was that non-invariance was around 30% when using criteria of effect size and invariance was at about 70%. This picture is certainly not as negative as earlier shown using inferential tests of significance in light of excessive power. Thus, there is a large degree of resemblance in the solutions between the years 2016 and 2017.

Table 6, shows the results from contrasting years 2017 and 2018. As shown in the table the number of significant tests using the logit was 13/24 (54.2%) and that number changed upwardly when applying an FDR correction using an alpha level of 0.001. Specifically, the number of significant tests was 14/24, that is, 58.3%. Using the probability difference tests, there were 16 significant tests, thus, non-invariance was at 66.7%, and using the *z*-test from the h-statistic (arcsine transformation) similar findings for non-invariance emerged (i.e., 13/24, 54.2%). When utilizing effect size conventions of the H-statistic, however, which reflects differences in proportions, there were 11 large effects (45.8%), 5 medium effects (20.8%), and 8 small effects (33.3%). Thus, a general conclusion from contrasting years 2016 and 2017 was that non-invariance was present to a large degree (for more than 50% of the items’ thresholds), leading to a conclusion of non-invariance.

Table 7 displays the findings from contrasting years 2016 and 2018 testing the hypothesis that invariance could be present with a lag of a single year. When contrasting the years 2016 and 2018, non-invariance was again evident. Specifically, the number of significant tests was 11/24, that is, 45.8%. Using the probability difference tests, there were 14 significant tests, thus, non-invariance was at 58.3%, and using the *z*-test from the h-statistic (arcsine transformation) similar findings for non-invariance emerged (i.e., 10/24, 41.7%). When utilizing effect size conventions of the H-statistic, however, which reflects differences in proportions, there were 10 large effects (41.7%), 3 medium effects (12.5%), and 11 small effects (45.8%). Thus, a general conclusion from contrasting years 2016 and 2018 was that non-invariance was present to a large degree (for more than 50% of the items’ thresholds), leading to a conclusion of subgroup non-invariance over time.

## Discussion

The present paper posed several research questions: For example: “Are there subgroups of individuals in which the student-level variables are combined to make up specific profiles?,” “Are these profiles meaningful and do they agree with the variable based analyses?,” “What is the prevalence/existence of those subgroups in the population?,” and “Are these profiles year-specific or invariant across years?” The answers are briefly provided below.

The first important finding was the existence of subgroups highlighting the influential role of the number of absences, and parents’ education on achievement. Generally speaking, classes with low numbers of absences and high levels in parents’ educational backgrounds were associated with elevated performance on the GAT science test. Interestingly, two above-average achieving classes were observed both having highly educated parents suggesting that parent education was a consistent positive covariate of academic achievement on the GAT science test. What differentiated, however, ideal students from average students was with regard to emitting absences. Both classes had highly educated parents but what made the difference in achievement, was the number of absences which was zero for the idea and highly achieving class but very prevalent in the average student class. Previous international studies confirmed the negative role of absences on student achievement (Smerillo et al., 2018). This finding is particularly more worrisome as there is a steady increase in the number of absences over the years. For example, based on the Department of Education data in the United States, the number of absences from high school increased by 6.8% in 2015–2016 compared to 2013–2014. This finding relating absences to academic achievement has potentially serious implications for educational policy through maybe enforcing stricter protocols on the number of absences allowed that still lead to degree attainment.

The positive role of parental education has been confirmed in earlier studies as well. For example, in a study by high school students whose parents had at least 1 year of college education had significantly higher scores compared to less-educated parents. The pathways to linking parents’ education to their student’s academic achievement have been through family processes (Conger et al., 2002) and the mediating roles of high income, high SES, high parental expectations (Epstein and Sheldon, 2002), high parental engagement and involvement, and many more (Gooding, 2001).

The second important finding was that two low achieving classes were observed that were distinct in males and females. Specifically, female students emitted many more absences compared to male students for approximately the same levels of performance; in fact, the achievement of female students was slightly higher compared to that of males, despite emitting twice as many absences. During the years 2016 and 2017, that was the sole difference among low achieving male and female students.

Another significant finding was that students’ age, which is customarily a positive predictor of academic achievement (Navarro et al., 2015), usually termed as the relative age effect (RAE, Musch and Grondin, 2001) proved to be a negative predictor in the present study. Possible explanations are the moderated effects of available time and the need to work, the lack of motivation, etc. Gender was not found to interact with age in producing the low achievement effect for older students.

A last important finding lies in the fact that the presence of mixtures of populations took into account the nesting of students within schools given that prior research has shown misrepresentations of subgroups, biased estimates of parameters and standard errors, and spurious groups that reflected artifacts of the analytical methodology (Vermunt, 2003, 2008; Meyers and Beretvas, 2006; Asparouhov and Muthen, 2008; Luo and Kwok, 2009; Chen et al., 2010; Park and Yu, 2016; Raykov et al., 2020). Accounting for nesting in the present study added confidence with regard to the generality of the observed subpopulations.

The present study’s findings are also limited for several reasons. First, invariance was tested although data came from cross-sectional samples. Indeed, it would be more appropriate to test invariance using a longitudinal design. Second, although a subsample of the population was employed, the sample size was still large enough so that some findings are likely reflective of Type-I errors. Third, informing the models for the variability across schools was taken into account, but additional nesting may have been more informative. For example, testing the effects of schools that are nested within urban versus rural regions. We deferred from including one additional level of structure because the number of regions in the Saudi Arabia Kingdom is twelve, and it is certainly small to warrant another level. This last limitation, however, can prove to be the necessary next step toward evaluating mixture models with additional layers of nesting, for which currently, there is very little research.

## Data Availability Statement

The raw data supporting the conclusions of this article will be made available by the authors, without undue reservation.

## Ethics Statement

The studies involving human participants were reviewed and approved by ETEC. The patients/participants provided their written informed consent to participate in this study.

## Author Contributions

GS designed the research project, performed the statistical analysis, and completed the original version of the manuscript. IT contributed to the development of the article, polished, revised, and approved the final version of the manuscript. KA-H collected the data, drafted parts of the “Method” sections, proofread the entire manuscript, and approved all parts of the written product. All authors contributed to the article and approved the submitted version.

## Conflict of Interest

The authors declare that the research was conducted in the absence of any commercial or financial relationships that could be construed as a potential conflict of interest.
