# Supplementary material for: Identifying Student Subgroups as a Function of School Level Attributes: A Multilevel Latent Class Analysis
Source: Front Psychol. 2021 Feb 26;12:624221. doi: 10.3389/fpsyg.2021.624221 (PMC7952435; doi:10.3389/fpsyg.2021.624221)
Supplement: Supplementary file 1 [file Table_1.DOCX]

**Supplementary Material**

Mplus syntax file for the class enumeration process and a test of a one-class solution. For testing a two class solution the statement CLASSES=c(1) needs to be substituted with CLASSES=c(2), etc., up to the number of classes requested. Each latent class solution represents a different input file.

TITLE: LCA

DATA: FILE IS rand1439a.dat;

VARIABLE: NAMES ARE k1-k64 age abs dad mom gat;

USEVARIABLES ARE k3 age abs dad mom gat;

CLASSES = c(1);

CATEGORICAL ARE k3 age abs dad mom gat;

cluster=k1;

MISSING ARE ALL(-9);

ANALYSIS: TYPE = Complex MIXTURE;

ALGORITHM=INTEGRATION;

STARTS = 500 100;

PROCESS = 2 (STARTS);

MODEL:

%OVERALL%

OUTPUT: SVALUES TECH11 TECH14;

For tests of invariance, the data of a specific year (e.g., 2017) were fit to a model in which that parameter estimates were fixed to values of a previous year (e.g., 2016). These parameter estimates could be easily obtained using the SVALUES command in the OUTPUT as shown above.
